# Supplementary material for: Very Early Response Evaluation by PET/MR in Patients with Lung Cancer—Timing and Feasibility
Source: Diagnostics (Basel). 2019 Mar 26;9(1):35. doi: 10.3390/diagnostics9010035 (PMC6468790; doi:10.3390/diagnostics9010035)
Supplement: Supplementary file 1 [file diagnostics-09-00035-s001.pdf]

Appendix 1

Response patterns as measured by diffusion weighted MRI (DWI) and FDG-PET in the individual patients. Results from DWI are presented as ADC_{mean} and ADC_{median} (mean and median apparent diffusion coefficient). Results from FDG-PET is presented as SUV_{max} and SUV_{peak} (maximum and peak standardized uptake value) together with $PERCIST_{ref}$ (SUV in a reference region in the liver measured according to the PERCIST recommendations[1]).

Patient 1

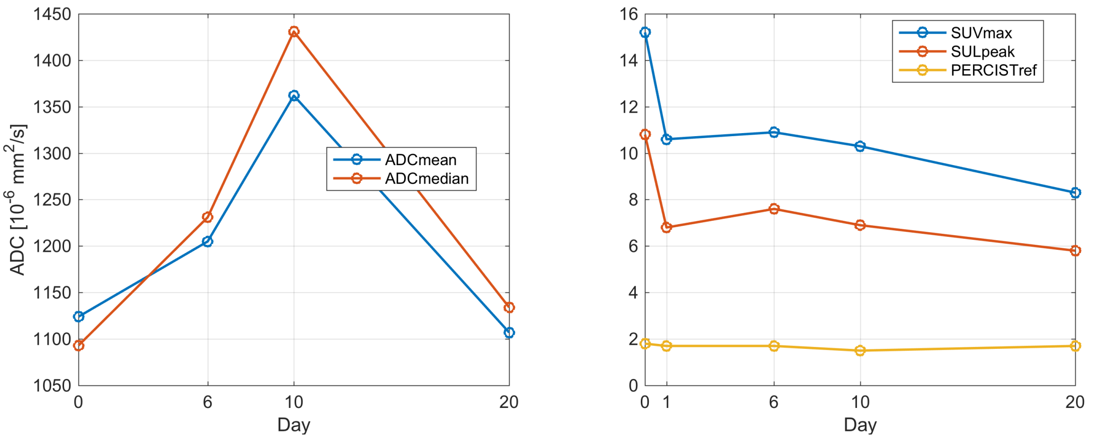

Patient 2

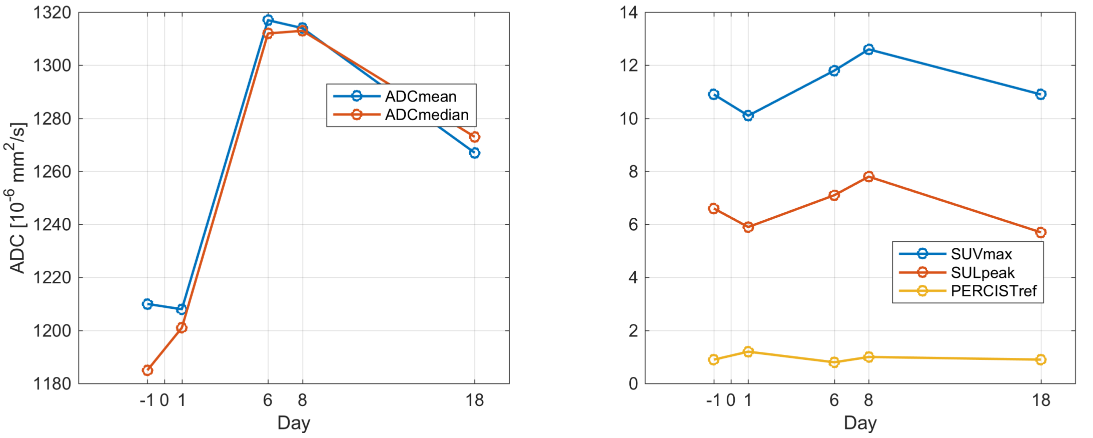

Patient 3

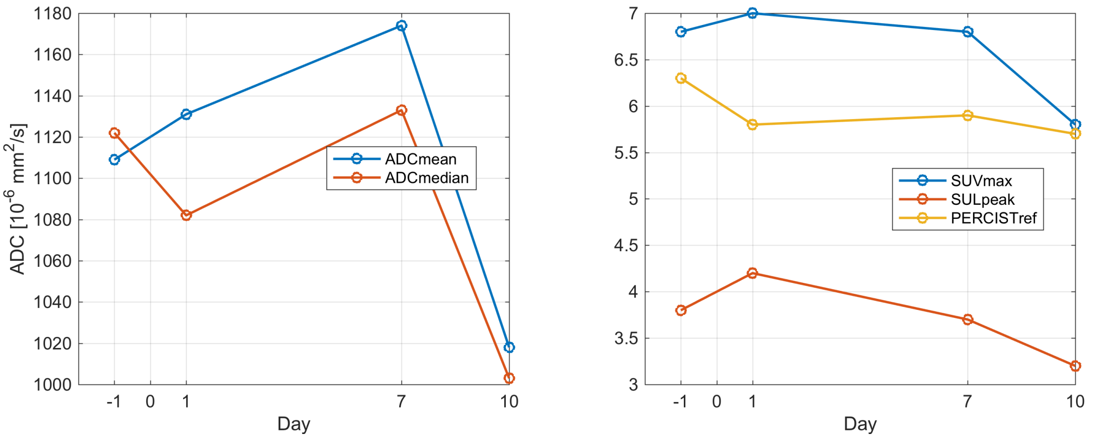

Patient 4

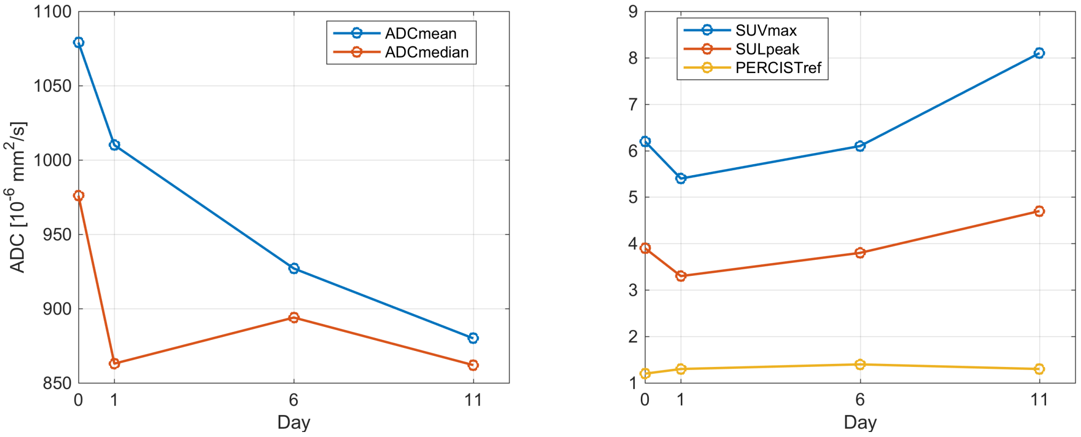

Patient 5

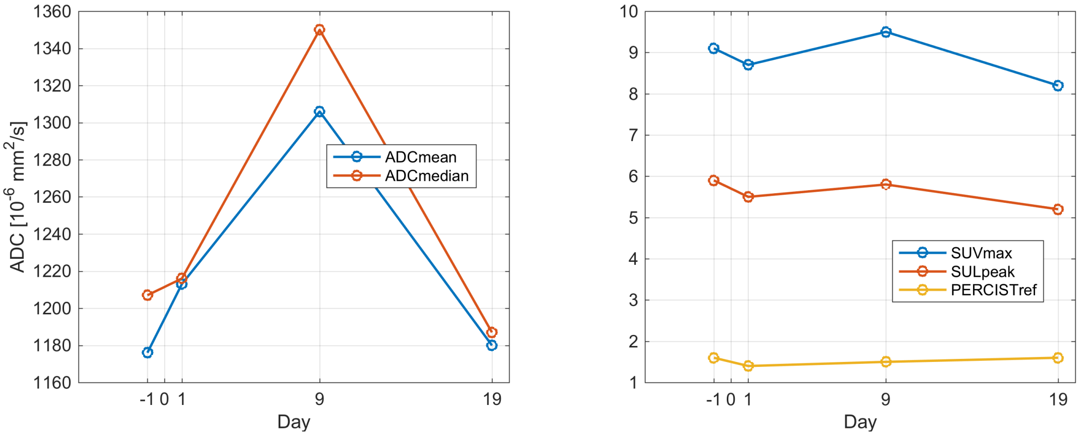

Patient 6

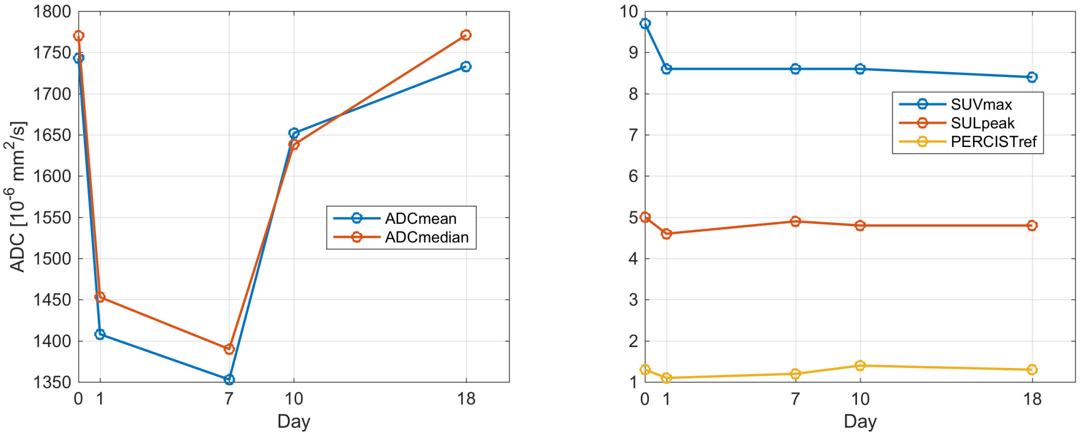

Patient 7

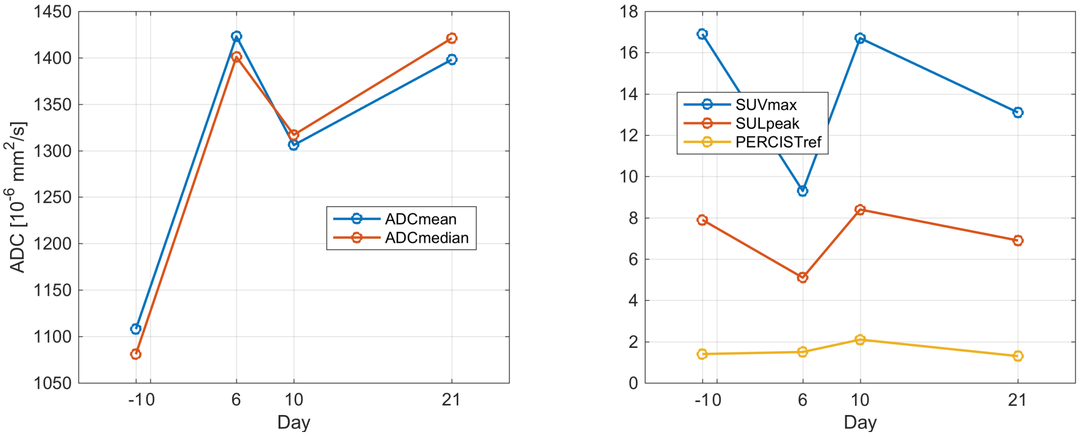

Patient 8

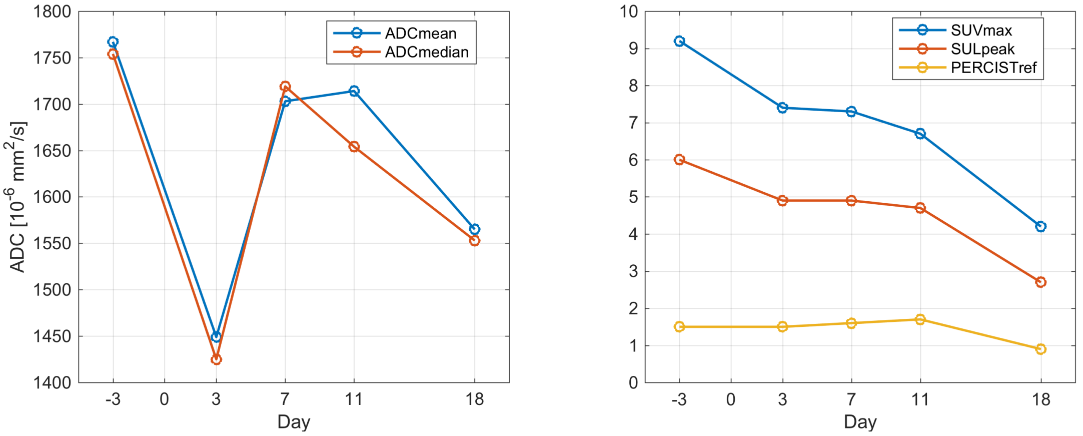

Patient 9

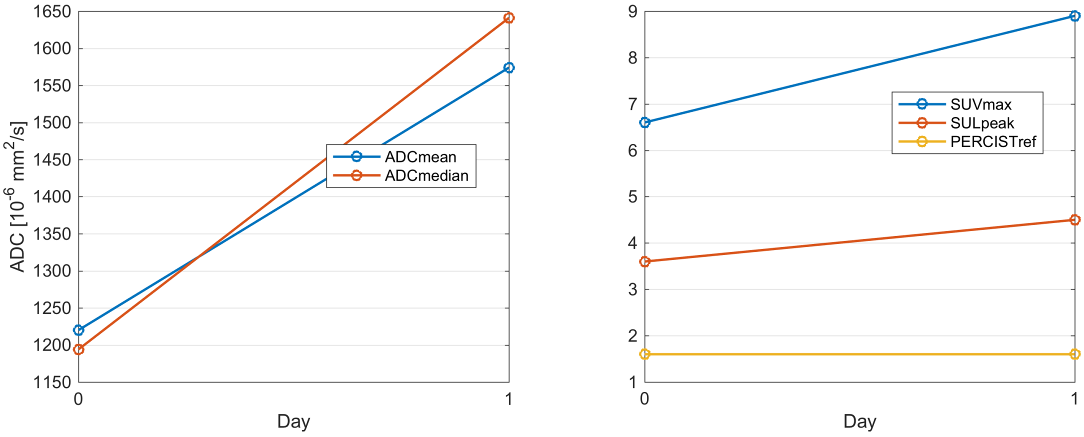

Patient 10

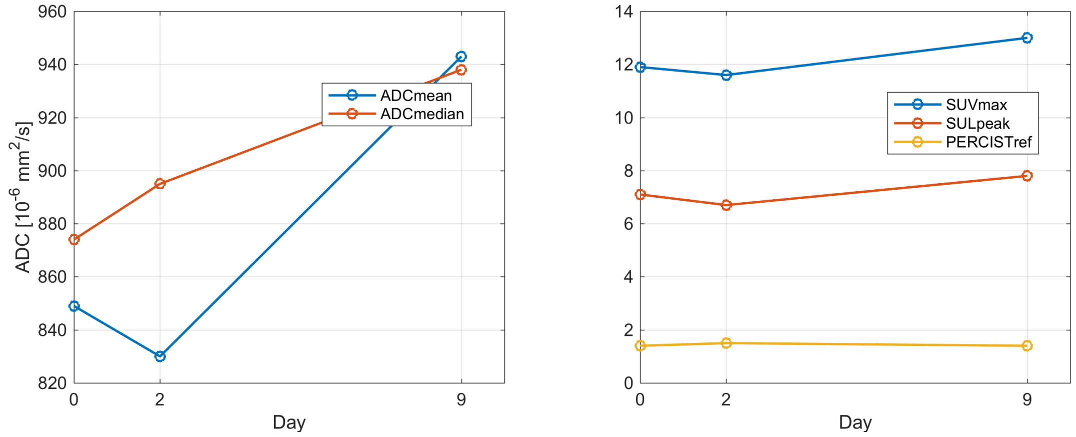

Patient 11

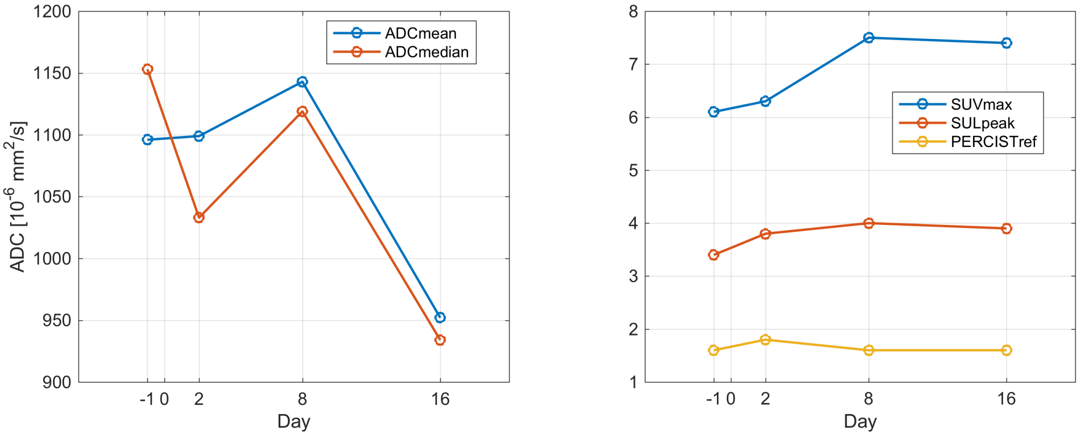

References

1. O, J.H., M.A. Lodge, and R.L. Wahl, *Practical PERCIST: A Simplified Guide to PET Response Criteria in Solid Tumors 1.0.* Radiology, 2016. **280**(2): p. 576-84.
